# Do smaller P300 amplitudes in schizophrenia result from larger variability in temporal processing?

**DOI:** 10.1038/s41537-024-00519-4

**Published:** 2024-11-07

**Authors:** Mareike Wilson, Ellen Joos, Anne Giersch, Anne Bonnefond, Ludger Tebartz van Elst, Lukas Hecker, Jürgen Kornmeier

**Affiliations:** 1 Department of Psychiatry and Psychotherapy, Medical Center—University of Freiburg, Freiburg, Germany; 2 Faculty of Medicine, University of Freiburg, Freiburg, Germany; 3 Institute for Frontier Areas of Psychology and Mental Health, Freiburg, Germany; 4 Faculty of Biology, University of Freiburg, Freiburg, Germany; 5 University of Strasbourg, INSERM U1329 (STEP), University Hospital of Strasbourg, Strasbourg, France

**Keywords:** Neuroscience, Psychology

## Abstract

The P3b is a prominent event-related potential (ERP) with maximal amplitude between 250 ms and 500 ms after the onset of a rare target stimulus within a sequence of standard non-target stimuli (oddball paradigm). Several studies found reduced P3b amplitudes in patients with schizophrenia compared to neurotypicals. Our work and the literature suggest that temporal imprecision may play a large pathophysiological role in schizophrenia. Here, we investigated whether reduced P3b amplitudes result from reduced neural activity (power) or temporal imprecision (inter-trial phase coherence; ITC) in delta and theta bands, using two EEG datasets from different studies with different oddball paradigms (Study 1: 19 patients with schizophrenia and 17 matched controls, Study 2: 26 patients and 26 controls). Both studies revealed typical P3b ERP components with smaller amplitudes in patients. Reduced ITC in patients was found in the delta band, which correlated with P3b peak amplitudes for all participant groups (ρ = 0.58–0.89). In Study 1, we also found significant differences between patients and controls in ITC in the theta band, which also correlated with P3b peak amplitudes (patients’ ρ = 0.64, controls’ ρ = 0.54). This was not found in Study 2. The results indicate that P3b amplitude reduction in patients with schizophrenia is linked to a reduction in temporal precision of neural activity. These results expand the notion of imprecision in temporal processing at phenomenological, psychological, and neurological levels that have been related to disturbances of the sense of self. They confirm that temporal imprecision may be more important than the reduction of neural activity itself.

## Introduction

Schizophrenia affects consciousness, social cognition, and communication, indicating deficits in high-level neural processing steps. The most prominent symptoms are hallucinations and delusions. Clinical diagnostics in psychiatry are based on subjective accounts collected during interviews with a psychiatrist. However, pathophysiology remains unclear and there is a need to establish a link with neurobiological mechanisms. A considerable number of EEG studies discuss the P3b being a potential objective physiological marker for schizophrenia related to clinical symptom severity across symptom categories, e.g., ref. ^1^.

The P3b is a prominent event-related potential (ERP) with maximal amplitude within a time window between 250 ms and 500 ms after the onset of a rare target stimulus within a sequence of frequent non-target stimuli (the oddball paradigm). The P3b can be evoked across stimulus modalities (visual, auditory, somatosensory) and response types (key press, counting)^2^. It can also be evoked by the absence of an expected stimulus within a sequence of regularly occurring stimuli. The P3b is typically most prominent at the Fz, Cz, and Pz EEG electrodes with a maximum at Pz^3^. P3b amplitudes increase with decreasing occurrence probability and increasing discriminability of the oddball stimulus (for reviews see refs. ^4–6^). Due to its late occurrence, its sensitivity to participants’ attentional state, and the context of the oddball stimulus rather than its physical features, the P3b is regarded as a cognitive and “endogenous” ERP component and a reliable measure of conscious access^7^.

The P3b effects are currently used in research as a potential objective measure of alterations in neural processing in patients with psychiatric disorders because of their large effect sizes. This is especially the case for patients with schizophrenia^1^. Reduced P3b amplitude in patients compared to neurotypicals has been regarded by some as one of the most robust physiological markers, with the quality of a genetic endophenotype^8^. A number of studies also report longer P3b peak times in patients compared to controls, e.g., ref. ^1^.

P3b differences between patients with schizophrenia and controls can be affected by stimulus and task variables. The size of the P3b amplitude difference between groups depends on target stimulus probability^9^, pitch disparity between auditory target and non-target stimuli^10^, response type (counting vs key press), and task^11,12^.

Another important and often ignored factor that may contribute, or even explain, P3b amplitude differences between patients with schizophrenia and controls, will be the focus of the present study.

The P3b is an ERP, which means that both target and non-target stimuli were repeatedly presented and the EEG trials were averaged over repetitions. The basic assumption underlying this averaging method is that the brain responses related to the processing of the presented stimulus, and particularly their timings, are mostly the same across repetitions. Therefore, peaks and troughs in the EEG traces occur at the same time and survive the averaging procedure. According to this logic, the ERP amplitude should decrease when the underlying processes are more variable in time. So, smaller P3b amplitudes in patients with schizophrenia can have at least three possible explanations:

1. Only a fraction of the processing units that typically contribute to the P3b amplitude are active in patients.
2. Some or all of the processing units are less active in patients.
3. Patients show more variability in the timing of the contributing neural processing units, resulting in a smaller amplitude of the P3b.

It is also possible that the P3b amplitude difference between patients and controls results from a mixture of (1–3). It is difficult to test for (1) and (2) because it is currently unclear how many and which brain processing units exactly contribute to the P3b. However, (3) is relatively easy to control for. EEG data series can be transformed into the frequency domain and decomposed into different frequency bands. The frequency analysis provides amplitudes and phases of the resulting signal frequencies. The phase information can then be used to investigate the timing of the neural activity, e.g., via the inter-trial coherence (ITC, as explained below).

Moreover, investigating variability in timing is relevant in light of recent studies stressing the importance of temporal imprecision. At the phenomenological level, psychiatrists have made a relationship between disorders of the sense of self and timing disorders^13–15^. Such a relationship has also been found in experimental approaches^16,17^. In a series of recent works, Northoff et al. have proposed that temporal imprecision at the neural level represents a basic disturbance in schizophrenia^18–20^. The authors measured neural activity at rest or before the onset of a stimulus, which entailed very long and variable durations. This may interfere with the patient’s ability to predict and adjust to external stimuli precisely in time. This is suggested by the relationship between the variability of response times and neuronal responses from trial to trial, i.e., a temporal imprecision from trial to trial^21,22^. Ultimately temporal imprecision may lead to time fragmentation at the subjective level and disturbances of the sense of self. In this framework, it is thus important to determine if markers like the P3b are related to time imprecision rather than a global decrease in neural activity.

In the present work, we compared the P3b between patients with schizophrenia and matched controls from two separate studies. We investigated whether P3b amplitude differences between patients and controls, reported from the literature, can be replicated. We further transformed the EEG data into the frequency domain to study whether the potential amplitude differences of the P3b are based on amplitude differences already present in single EEG trials or on equal amplitudes but larger temporal variability across single trials in the patient group.

## Methods and materials

### Participants

Table 1 depicts all the participant information from both studies. Study 1 had 19 patients with schizophrenia and 17 matched controls and Study 2 had 26 patients with schizophrenia and 26 matched controls. Also depicted are gender, age, dose of chlorpromazine equivalents (mg/d), disease duration in years, and clinical evaluation scores. In Study 1, patients had their clinical evaluation done with a scale for the assessment of positive symptoms (SAPS)^23^ and a scale for the assessment of negative symptoms (SANS)^24^, and in Study 2, the Positive and Negative Syndrome Scale (PANSS)^25^ was used. Two patients in Study 2 did not have PANSS scores. To allow for a comparison between studies, the SAPS/SANS scores from Study 1 were converted into PANSS scores. Informed consent was obtained from all participants and the studies were approved by the local ethics committee (CPP IV Est). Psychiatric diagnoses were established by a senior psychiatrist from the department. Diagnoses fulfilled the Diagnostic and Statistical Manual of Mental Disorders, Fifth Edition^26^, criteria for a diagnosis of schizophrenia. The patients were all outpatients. Exclusion criteria for patients and controls were intake of benzodiazepines, history of alcohol and drug dependency, neurological and medical pathologies, disabling sensory disorder, and general anesthesia in the 3 months prior to testing. Additionally, the exclusion criterion for controls was psychotropic medication in the 3 weeks prior to testing.

**Table 1:** Demographic and clinical data of the participants in Study 1 (first two columns) and Study 2 (second two columns).

	Study 1		Study 2	
	Patients (*n* = 19)	Controls (*n* = 17)	Patients (*n* = 26)	Controls (*n* = 26)
Gender (M/F)	13/6	11/6	17/9	17/7 (2 unknown)
Age	36.79 (±8.64)	36.88 (±9.02)	39.15 (±8.48)	37.62 (±7.85)
Dose of chlorpromazine equivalents, (mg/d)	295.88 (±181.62)	–	242.96 (±143.35)	–
Disease duration in years	13.06 (±10.26)	–	15.39 (±7.73)	–
SAPS total score	20.74 (±19.48)	–	–	–
SANS total score	33.79 (±22.79)	–	–	–
PANSS total positive score	14.95 (±4.42)*	–	16.38 (±4.84)	–
PANSS total negative score	18.63 (±6.32)*	–	18 (±5.5)	–
PANSS total general score	31.11 (±8.03)*	–	31.96 (±9.98)	–
PANSS total global score	64.68 (±14.6)*	–	66.33 (±16.99)	–

Depicted are the group means ± standard deviations (except for gender). The PANSS scores for Study 1 were converted from the SAPS/SANS data collected initially (this is depicted by a *) to allow for a comparison between Study 1 and Study 2. Study 2 does not have SAPS/SANS scores.

### Stimuli and procedure

**Fig. 1:**
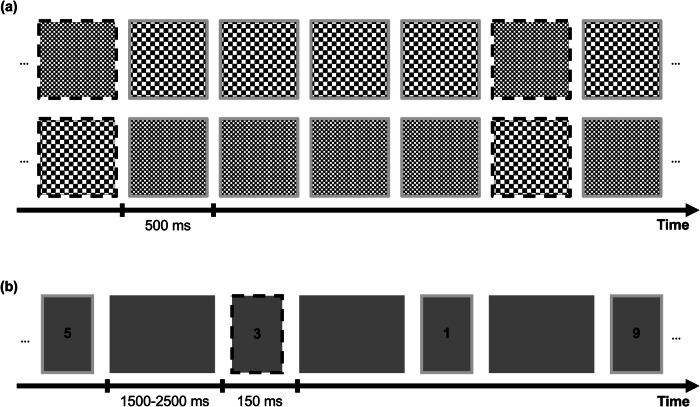
Paradigms for studies 1 and 2. **a** Checkerboard oddball paradigm and **b** number oddball paradigm. In **a**, the top row corresponds to the Small Rare Condition, and the bottom row to the Large Rare Condition. Marking of the oddballs by a dashed black frame is for demonstration purposes in the current figure. The oddball occurred after every fifth stimulus. Each checkerboard was presented for 500 ms and participants had to count the occurrence of the rare stimuli and report this number at the end of an experimental block. As seen in **b**, participants were either presented with a Go or NoGo trial (black dashed lines, oddball) for the second study. During a Go trial, participants were asked to press a button if a number was presented that was not 3. During a NoGo trial, participants were presented with the number 3 (oddball stimulus) and needed to refrain from pressing the button. The stimuli were presented for 150 ms. The interstimulus interval was presented for 1.5–2.5 s^21^.

For Study 1, stimuli were coded in Python using Psychopy v1.82^27^. Participants were presented with two differently sized checkerboards in an oddball paradigm (see Fig. 1a). The checkerboards, presented for 500 ms, were frequent (standard) or rare (oddball), consisting of two conditions (large rare/small frequent or small rare/large frequent). The conditions were presented separately and the order was pseudo-randomized. Every fifth stimulus was an oddball and participants were asked to silently count the number of oddballs, reporting this number at the end of the condition.

In Study 2, participants were presented with digits between 1 and 9 (Fig. 1b). Each digit was presented with equal probability and for a duration of 150 ms. Between each stimulus, there was an interstimulus interval with a dark screen that had varying lengths, ranging from 1.5 s to 2.5 s. Participants were asked to respond via button press if a 1, 2, or 4–9 occurred (Go-trials, standard) and to refrain from pressing a button when a three occurred (NoGo-trial, oddball). The data from this study have already been published by Chidharom et al. with a different experimental question and separate analysis^21^.

### EEG recording and pre-processing

Both datasets were recorded using a 64 Ag/AgCl BioSemi ActiveTwo System (Amsterdam, Netherlands). Electrodes were mounted on a cap following the standard 10–20 system^21^. The data from Study 1 had a sample frequency of 2048 Hz and the data of Study 2 had 512 Hz.

All pre-processing was done using the MNE-Python package (version 1.4.2)^28^ in Python 3.9.6. An Independent Component Analysis (ICA) was calculated to detect eye blinks using standard MNE procedure. A correlation was calculated between data from the FP1 electrode and the Independent Components to determine which components most correlated with eye blinks. A component would be labeled as an eyeblink if the *z*-score was over 3.0 (default MNE procedure and values). The eyeblink component was then removed from the data. The FP1 electrode was bandpass filtered from 1 Hz to 10 Hz before correlation with the ICA. After correction for eye blink artifacts, the original data was then offline band-passed from 0.01 Hz to 30 Hz.

Using the RANSAC (Random Sample Consensus) algorithm from the autoreject package (version 0.4.2)^29,30^ bad channels were detected. This was done by calculating the correlation between a channel and its neighbors. Channels that had a low correlation (correlation of less than 0.75) were labeled as bad and interpolated using the data from neighboring channels. The data was re-referenced at the TP7 and TP9 electrodes and baseline corrected, using the average amplitude in a time window between 60 ms before stimulus onset and 40 ms after stimulus onset. Lastly, epochs that had signals over ±100 µV (in Study 1) and ±150 µV (in Study 2) were rejected. For Study 1, if participants had both conditions (large rare and small rare), the condition presented first was used for analysis. If participants only had one condition, the only condition presented was used. For specific participants, the preprocessing pipeline was changed slightly, to allow for enough trials after preprocessing. A description of what was done for which participants can be seen in Supplementary Materials, Table 1. Altogether, there were a minimum of 44 trials per person for the analysis of Study 1 and 43 trials for Study 2.

### Data analysis

Data analysis was done using the same MNE package version and Python version as for preprocessing.

#### ERP analysis

Single-trial EEG data for the oddball and standard stimuli at electrode Pz were separately averaged to ERPs. For each experiment, the standard ERPs were subtracted from the oddball ERPs on the group level in order to isolate the P3b and get a temporal region of interest (tROI). Using the P3b in the grand mean difference trace, we defined a tROI with the full-width half maximum method using the control group difference trace. The tROI was further used to isolate the P3b peak in single-participant ERPs. The individual peaks were calculated by using the SciPy signal function^31^, finding peaks in the tROI and choosing the peak with the highest amplitude. The latency of the highest amplitude was determined, resulting in a peak amplitude and latency for every participant.

#### Time-frequency analysis

The time-frequency analysis was done using a time-resolved Morlet wavelet analysis. From this analysis, phase, and power were extracted from the oddball trace (not the oddball-standard difference trace). The total power was calculated for every single trial. This power was then averaged across trials. Total power includes activity phase-locked to stimulus onset, as well as non-phase-locked background activity. Total power differs from evoked (phase-locked activity) and induced power (only non-phase-locked background activity). For visualization purposes, the MNE Morlet wavelet analysis output was normalized by dividing every value by 11.82^2^, resulting in total power values in µV^2^.

The phase information was used to calculate the inter-trial coherence (ITC). The phases for a specific time window are calculated across trials.

$${{ITC}}\left(f,t\right)=\left|\frac{1}{n}\mathop{\sum }\limits_{k=1}^{n}\frac{{F}_{k}(f,t)}{{{{|}}F}_{k}(f,t){{|}}}\right|$$

where *n* is the number of trials and *F*_*k*_(*f*, *t*) is the time-frequency transform of the signal (complex number) of trial *k* at frequency *f* and time *t*. The time-frequency transform of the signal is divided by its absolute value. The mean of the normalized values for every trial is then calculated. The absolute mean value is referred to as the ITC^28,32^ and is a measure of phase variability across trials.

If the phases across trials are exactly the same, the ITC is 1 and if the phases across trials are maximally different, it is 0.

To calculate total power and ITC in delta (2–4 Hz) and theta (4–8 Hz) bands, the median across values at the P3b peak amplitude time point for each frequency within a band was calculated. The motivation behind not including 1 Hz in the delta band was because the trial lengths were one second long and therefore would not yield accurate results at such a low frequency. Boxplots were used to summarize the distribution of median delta and theta total power and ITC across participants for the P3b peak amplitude time point. In each boxplot, the central line represents the median, and the edges are the first and third quartiles, while the whiskers extend to show the rest of the distribution (without outliers).

#### Correlations

Individual P3b peak amplitudes were correlated with ITC values as the final hypothesis-driven analysis. Additionally, as an explorative analysis, individual P3b peak amplitudes and ITC values were correlated with clinical evaluation scores, amongst other variables. As previously mentioned, to pool both studies, SAPS/SANS clinical evaluations from Study 1 were converted to PANSS scores. For patients who participated in both Studies 1 and 2, only ITC values for Study 1 were used (two participants). Additionally, two patients did not have PANSS scores and therefore could not be used in the correlation analysis. This resulted in a total of 41 patients. Variables that were correlated with P3b peak amplitudes and ITC values for delta and theta separately included: age, neuroleptics equivalent dose (mg/day), PANSS total positive, PANSS total negative, PANSS total general, PANSS total global, disorganized P2 N5 G10 G11, disorganized P2 P4 N5 N7, negative N1 G7 G13 G16, and positive P1 P3 G9.

#### Statistical analysis

A one-sided Wilcoxon rank-sum test^33,34^ was performed to compare peak P3b amplitudes, latency, total power, and ITC values between controls and patients. Correcting for multiple testing was done using the Bonferroni-Holm method^35^, and was done for three tests, due to the two hypotheses we had, (1) ERP amplitude reduction in patients is due to signal strength or (2) ERP amplitude reduction is due to temporal jitter along with (3) the determining analysis correlating ERP amplitude with ITC. All correlations were done using the Spearman correlation^36^.

## Results

### Study 1

**Fig. 2:**
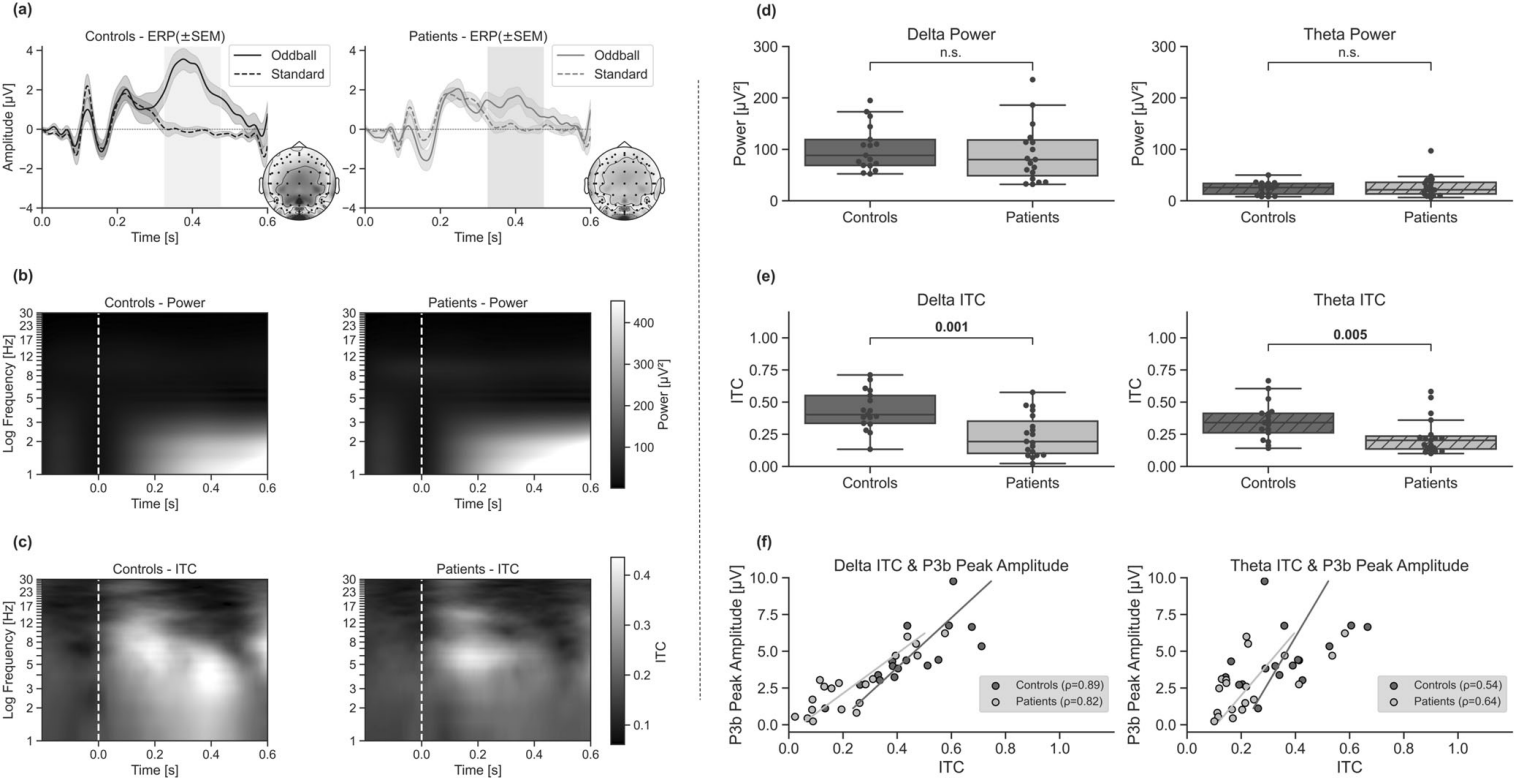
Results of Study 1 at electrode Pz (ERP, power, and ITC). Depicted in (**a**) is the grand mean ERP (in µV) ± standard error of the mean (SEM). Dashed lines depict the standard stimulus, while the solid lines are the oddball stimulus. The gray rectangles show the time window of the P3b analysis. Voltage maps right of each ERP graph depict interpolated scalp amplitude distributions at the time point of the maximal P3b amplitude of the oddball ERP trace (at around 0.4 s). **b** Shows the total power (µV^2^) of the oddball trace where white signifies high total power and black has low total power. Depicted on the *y*-axis is the frequency (Hz) log-scaled. And (**c**) shows the ITC results, calculated on the oddball trials, where white depicts a higher ITC and black a lower ITC. The two columns show the different participant groups (the left column matches controls, the right column patients with schizophrenia). The *x*-axes of all three subplots depict time in seconds, with 0 s indicating stimulus onset. Also depicted are a single participant (**d**) total power and (**e**) ITC only in delta and theta bands (including *p*-values), and (**f**) the correlation between ITC (*x*-axis) and P3b peak amplitude in µV (*y*-axis). Dark gray/light gray icons: individual data from the matched controls/patients with schizophrenia. The graphs indicate a positive correlation between P3b amplitude and the degree of temporal precision across single trials (ITC) (controls’ delta correlation *p*-value = 3 × 10^−6^, patients’ delta correlation *p*-value = 2 × 10^−5^, controls’ theta correlation *p*-value = 0.01, patients’ theta correlation *p*-value = 0.002).

As seen in Fig. 2a, a P3b can be seen in the matched controls but not in the patients with schizophrenia. The amplitude difference is statistically significant (statistic = 2.6, *p* = 0.005, Cohen’s *d* = 0.9) while the latency is not (*p* = 0.13). Interestingly, total power calculations reveal a different picture, with slightly higher amplitudes at lower frequency bands (delta and theta) in patients compared to controls (Fig. 2b). This difference, however, is not significantly different between groups (delta *p* = 0.24, theta *p* = 0.56; Fig. 2d).

ITC is higher in controls compared to patients, with most coherence in lower frequency bands (delta and theta). ITC is highest at 0.2 s after oddball stimulus onset and around the P3b time window (around 0.4 s) for controls. For patients, only one ITC peak at around 0.2 s after stimulus onset can be observed (Fig. 2c), indicating less temporal precision in the P3b time window. The larger ITC in controls compared to patients is statistically significant in the delta band (statistic = 3.06, *p* = 0.001, Cohen’s *d* = 1.2) and in theta band (statistic = 2.6, *p* = 0.005, Cohen’s *d* = 0.8) (Fig. 2e).

As seen in Fig. 2f, the correlation between P3b peak amplitudes and delta ITC is statistically significant in both controls (ρ = 0.89, *p* = 0.000003) and patients (ρ = 0.82, *p* = 0.00002). The correlation between theta ITC and P3b amplitudes for controls (ρ = 0.54, *p* = 0.01) and patients (ρ = 0.64, *p* = 0.002) is also significant.

### Study 2

**Fig. 3:**
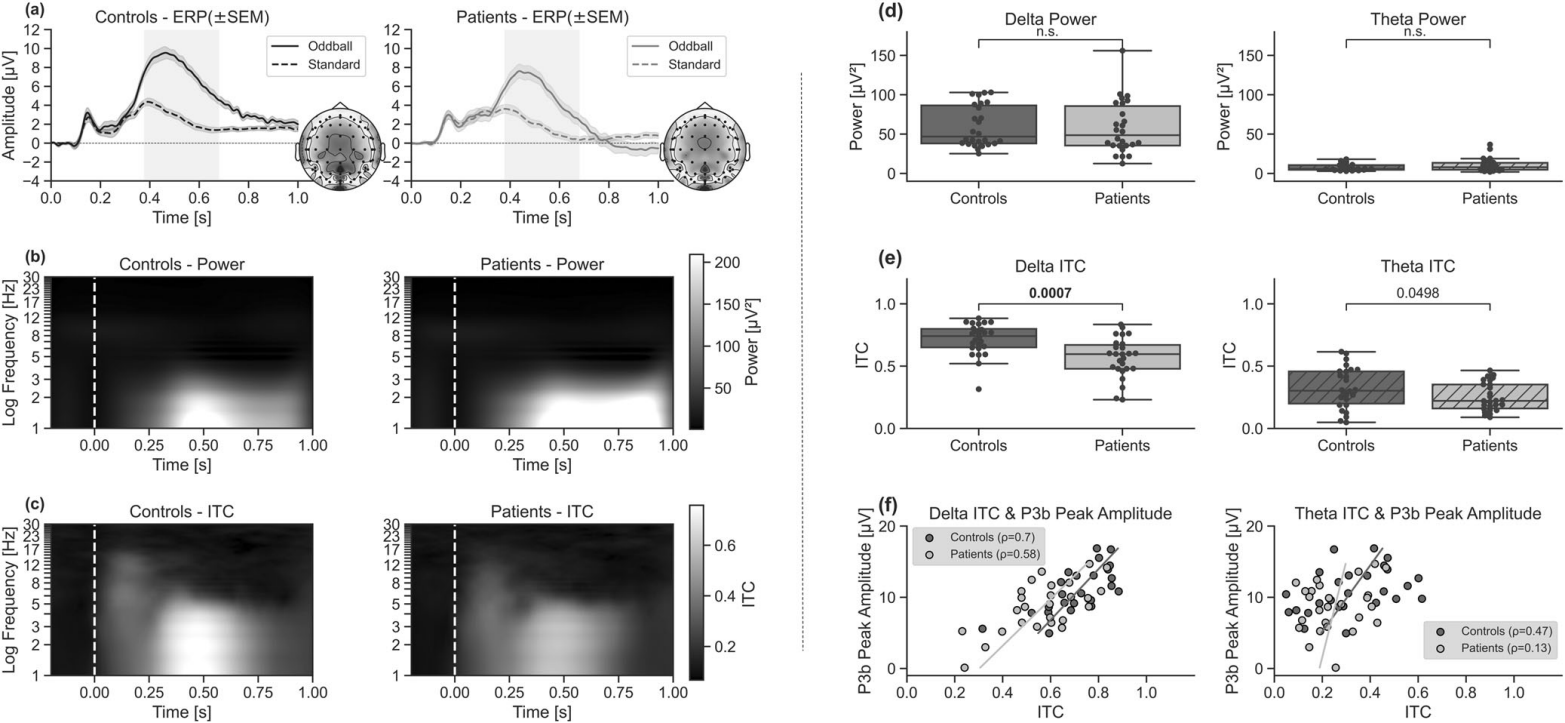
Results of study 2 at Electrode Pz (ERP, power, ITC). Depicted in (**a**) is the grand mean ERP (in µV) ± standard error of the mean (SEM). Dashed lines depict the standard stimulus, while the solid lines are the oddball stimulus. The gray rectangles show the time window of the P3b analysis. Voltage maps right of each ERP graph depict interpolated scalp amplitude distributions at the time point of the maximal P3b amplitude of the oddball ERP trace (at around 0.45 s). **b** Shows the total power (µV^2^) of the oddball trace where white signifies high total power and black has low total power. Depicted on the *y*-axis is the frequency (Hz) log-scaled. And (**c**) shows the ITC results, calculated on the oddball trials, where white depicts a higher ITC and black a lower ITC. The two columns show the different participant groups (the left column matched controls, the right column patients with schizophrenia). The *x*-axes of all three subplots depict time in seconds, with 0 s indicating stimulus onset. Also depicted are a single participant (**d**) total power and (**e**) ITC only in delta and theta bands (including *p*-values), and (**f**) the correlation between ITC (*x*-axis) and P3b peak amplitude in µV (*y*-axis). Dark gray/light gray icons: individual data from the matched controls/patients with schizophrenia. The graphs indicate a positive correlation between P3b amplitude and the degree of temporal precision across single trials (ITC) (controls’ delta correlation *p*-value = 1 × 10^−4^, patients’ delta correlation *p*-value = 0.001, controls’ theta correlation *p*-value = 0.007, patients’ theta correlation *p*-value = not significant).

In Fig. 3a, a P3b can be observed with higher amplitude for the oddball compared to the standard stimulus in both groups. The P3b amplitude in the patient group is slightly lower compared to the control group (statistic = 1.96, *p* = 0.03, Cohen’s *d* = 0.7) but latency does not differ between groups (*p* = 0.42). Focusing on the frequency transformed data, the maximum total power in both groups seems to be around 0.5 s after stimulus onset and does not significantly differ between groups (delta *p* = 0.27, theta *p* = 0.81; Fig. 3b, d).

The ITC is higher in controls compared to patients and both have the highest ITC around 0.5 s after stimulus onset (Fig. 3c). There are significant differences between ITC in the two groups in the delta band (statistic = 3.4, *p* = 0.0007, Cohen’s *d* = 1) and marginally significant differences in the theta band (statistic = 1.65, *p* = 0.0498, Cohen’s *d* = 0.5) as seen in Fig. 3e. For single participant examples of ERPs and single trials, see Supplementary Materials, Fig. 1.

When correlating P3b amplitudes with the delta ITC, there is a significant correlation in controls (ρ = 0.7, *p* = 0.0001) and patients (ρ = 0.58, *p* = 0.001). In the theta band, there is a significant correlation in the controls (ρ = 0.47, *p* = 0.007) but not in the patients (*p* = 0.26) (Fig. 3f).

### Correlations with demographic and clinical data

A correlation between delta and theta ITC values and PANSS scores, age, neuroleptics, and equivalent dose (mg/day) showed no significant results. Additionally, there was no significant correlation between demographic and clinical data and P3b peak amplitudes (Supplementary Materials, Table 2).

## Discussion

In EEG data from two separate oddball studies, we found smaller P3b amplitudes in patients with schizophrenia compared to matched controls, which confirms numerous previous findings (see introduction). Analysis of theta and delta bands of frequency-transformed EEG data in the respective time window revealed no significant difference in total power. However, patients displayed significantly smaller inter-trial coherence (ITC) in the delta band than controls. These findings are accompanied by a high correlation between delta ITC and P3b peak amplitude across participants.

### Relation of current findings to literature

There were three options to explain an observed reduction in P3b amplitude in patients with schizophrenia compared to matched controls. The first option assumes a reduction in a number of active processing units, and the second a reduced intensity of their activity. Our results point towards option three, a loss in temporal precision of neural activity (with respect to stimulus onset) across repetitions in patients, resulting in a reduction of the averaged P3b amplitude. One potential reason why we find stronger effects in the delta band as compared to the theta band might be due to the fact that delta frequencies seem to play a bigger role in the parietal P300 signal than theta frequencies^37^.

The literature describes the reduction in P3b amplitude as one of the most substantial physiological markers in schizophrenia research^8^ (also see “Introduction”). Among all EEG studies with a focus on the P3b in patients with Schizophrenia Spectrum Disorders (SSD), we only found six studies with a specific focus on temporal precision of the P3b. Those studies looked at how the precision of the P3b across trials affected power and/or phase in delta and theta frequency bands in the P3b spatiotemporal region of interest. Five studies used auditory and only one visual stimulation. The results from the auditory oddball studies are considerably heterogeneous: in patients with schizophrenia compared to neurotypical controls, Ford et al.^38^ found fewer single EEG trials containing a P3b, smaller amplitudes in trials where they identified a P3b, and larger variability of P3b latencies across trials. In a follow-up study^39^, they were able to confirm the finding of smaller P3b amplitudes in patients together with a smaller delta and theta ITC. Like in the present study, they did not find a difference in total delta and theta power between patients and controls. Röschke and Fell^40^, while not looking at ITC specifically, found smaller P3b amplitudes and smaller power in the delta frequency. Doege et al.^41^ reported smaller P3b amplitudes in patients, compared to controls, together with smaller evoked and induced delta and theta power. The study from Shin et al.^42^ revealed smaller P3b amplitudes and both smaller total delta and theta power and ITCs. Almeida et al.^43^ did not find a P3b amplitude difference between patients and controls; however, their regression analysis revealed a high correlation between P3b amplitude on one hand and delta total power and ITC on the other hand. The last and most recent auditory oddball study by Wu et al.^44^ focused on individuals with clinically high risk for psychosis. The results of those individuals are especially interesting, if one considers that the EEG measures may represent biomarkers of the conversion to psychosis. Wu et al. found reduced P3b amplitudes together with decreased delta power and ITC in a subgroup of their patients compared to the controls. Roth et al.^45^ investigated the P300 with similar methods to ours and also found increases in latency variability at electrode Fz and amplitude variability at electrode Pz in the schizophrenia group. The only oddball study with visual stimulation and EEG frequency analysis, we found, is from Ergen et al.^46^. The authors report reduced P3b amplitudes and evoked delta power in patients with schizophrenia, but no difference in the total delta power. While this result points to larger temporal variability in the patients, the authors did not explicitly analyze the phase data.

The discrepancies in results between the auditory oddball studies and the current results may, at least partially, be explained by the different stimulus modalities. The discrepancies within the auditory oddball studies may be explained by specific differences in the paradigms (e.g., the probability of the oddball) and/or differences in patient sub-populations in the respective studies. A detailed discussion about this, however, is beyond the scope of our present study, given the overall confirming evidence. Moreover, the results from the single visual oddball study^46^ align with the present results.

The wide range of results, suggests that the big picture is more complex and that it is difficult to draw conclusions from different studies. Therefore, it is notable that, despite having data collected from two different oddball studies (with different stimuli and different tasks), we are able to show consistent ITC results across paradigms, for a total of 45 patients. The results from Study 2 also fit other analyses done on the same data. Chidharom et al.^21^ found that patients with schizophrenia also exhibit intraindividual variability of reaction time which is in part linked to attentional fluctuations and discuss the correlated underlying neural processes.

### Neural mechanisms underlying present findings

Having a closer look at the temporal dimension of brain processing may help to better understand the present results. Several EEG studies report a very close relationship between the P3b signature and activity in the theta and delta frequency bands e.g., refs. ^47–49^. While the following is speculative, current models assume that neural activity measurable at EEG scalp electrodes necessitates synchronized oscillatory activity of millions of underlying neurons, sometimes called neural assemblies, on a milliseconds time scale. Only their synchronized activity allows addition rather than mutual extinction of potentials with positive and negative signs and thus the visibility of this neural activity at scalp EEG electrodes^50–53^.

These considerations make frequency analysis an elegant method to investigate the origin of P3b amplitude reduction in patients with schizophrenia. Applying this technique strongly indicates that the amplitude reduction in the ERP in the patient groups most probably results from a larger temporal variability of the neural processes underlying the P3b across single trials. Moreover, the lack of differences in the total power in the respective frequency bands between patients and controls indicates that after taking into account this temporal variability, the difference in power between groups disappears.

Precision in neural activity in the milliseconds time scale is necessary to allow for an increase in total power in the P3b spatio-temporal window in single trial data. The fact that there is similar total power in theta and delta frequency bands between groups, indicates this type of precision to be intact in patients. It may reflect synchronized activity within neural assemblies, where the time reference for the synchronization during a trial is unknown.

Our finding of smaller ITC, on the other hand, indicates an imprecision across stimulus repetitions and thus at a larger time scale in the range of tens of milliseconds. ERP amplitudes, to a large degree, depend on synchronized activity to certain events as a time reference (in our case, the onset of the oddball stimulus).

There is a discussion about whether an evoked potential is an additive stimulus-related (evoked) response, independent of ongoing neural background activity, or whether it reflects a reset of ongoing neural oscillations^54^. Several findings indicate that both options are realized in the brain and reflect either early sensory, bottom-up activity (additive responses) or later and more cognitive top–down activity (resetting approach), e.g., refs. ^53,55^. Along the spatial and temporal hierarchy of the perceptual system, the P3b is at a relatively late^56–60^ and probably at an integrative stage between sensory and motor processing, e.g., ref. ^61^. and close to the time point of conscious access^7,58^. Due to the fact that the P3b occurs rather late, the temporal imprecision of the evoked P3b EEG response of patients across oddball stimulus repetitions thus seems to reflect imprecision of an additive or integrative cognitive response (top–down processes), rather than imprecision in the reset of oscillatory activity. How exactly this temporal imprecision comes about, however, is unclear.

In summary, similar to the studies of Northoff et al.^18–20,22^., we observed a temporal imprecision rather than a global decrease of neural activity in relation to the processes underlying the P3b. Our results are consistent with those prior studies but also extend them by suggesting a relationship between temporal imprecision and an evoked potential that has been widely studied in patients with schizophrenia. Whether this imprecision is related to imprecision in the preparation to stimulus processing remains to be seen. Like in Northoff et al.’s studies, we did not find correlations with clinical evaluations, and like in those studies we can note that these evaluations are not well designed to measure the disorders of the sense of self. It can be noted, though, that the P3b has been related to conscious access^62^ and may be one of the mechanisms mediating the temporal fragmentation that is reported by patients. Being able to time one’s own thoughts with precision and regularity, as well as timing reactions to the environment, is necessary to feel immersed in the environment, i.e., to predict and follow the flow of events fluently. Conversely, we can suggest that the temporal imprecision of the P3b may reflect the lack of immersion as frequently observed in SSD. It would be interesting to know if in pathologies also showing a decreased P3b amplitude (see e.g., ref. ^63^), the mechanisms leading to this decrease are similar or differ from schizophrenia. Overall, the results suggest that the interpretation of the reduction in P3b (and potentially other ERP components) amplitudes needs to be re-evaluated in light of the timing impairments.

## Supplementary information

Supplementary Material

## Data Availability

The data that support the findings of this study are available from the corresponding author upon reasonable request.

## Code availability

The code used in this analysis will be available to download under the following link: https://osf.io/sh2g7/?view_only=9fe9cda42aaf4c36a22cef8e2365ce22.
