# Supplementary material for: Do smaller P300 amplitudes in schizophrenia result from larger variability in temporal processing?
Source: Schizophrenia (Heidelb). 2024 Nov 7;10(1):104. doi: 10.1038/s41537-024-00519-4 (PMC11544218; doi:10.1038/s41537-024-00519-4)
Supplement: Supplementary file 1 — Supplementary Material [file 41537_2024_519_MOESM1_ESM.docx]

# Supplementary Material

| **Participant** | **Group** | **Changes to pre-processing** |
| --- | --- | --- |
| SH | Patients | Changed the eyeblink ICA correlation threshold to 4.0 (z-score) |
| S7 | Controls | Changed the eyeblink ICA correlation threshold to 4.0 (z-score) |
| SG | Controls | Changed amplitude rejection threshold to ± 150µV |
| SL | Controls | Due to artifacts on all channels the condition recorded later was used |
| ST | Controls | Changed the eyeblink ICA correlation threshold to 4.0 (z-score) |

**Supplementary Table 1.** **Pre-processing deviations from the standard pipeline.** Information about participants whose preprocessing pipeline differed from the norm for Study 1.

**Supplementary Figure 1. Four exemplary single participant results.** The left column (a and b) shows two control participants and the right (c and d), two patients. The first row (a and c) shows results from Study 1 and the second (b and d) from Study 2. The top plots in each subplot show the single trial amplitudes in µV, with time in seconds on the x-axis and single trials on the y-axis. The lighter the colour, the higher the amplitude. The plot below shows the single participant ERP (±SEM shaded in gray), with time in seconds on the x-axis and amplitude in µV on the y-axis. Time point zero is stimulus onset.

| **Variable Correlated with EEG Variable** | **Delta ITC Spearman Correlation (ρ, p-value)** | **Theta ITC Spearman Correlation (ρ, p-value)** | **P3b Amplitude Spearman Correlation (ρ, p-value)** |
| --- | --- | --- | --- |
| Age | 0.035, 0.83 | -0.0044, 0.98 | 0.0086, 0.96 |
| Neuroleptics equivalent dose (mg/day) | -0.0044, 0.98 | 0.13, 0.4 | -0.066, 0.68 |
| PANSS total positive | 0.12, 0.44 | 0.24, 0.13 | 0.15, 0.35 |
| PANSS total negative | 0.056, 0.73 | 0.14, 0.38 | 0.11, 0.51 |
| PANSS total general | 0.063, 0.69 | 0.23, 0.15 | 0.086, 0.59 |
| PANSS total global | 0.071, 0.66 | 0.19, 0.23 | 0.11, 0.49 |
| Disorganized P2 N5 G10 G11 | -0.058, 0.72 | 0.20, 0.22 | -0.056, 0.73 |
| Disorganized P2 P4 N5 N7 | -0.030, 0.85 | 0.18, 0.25 | 0.021, 0.9 |
| Negative N1 G7 G13 G16 | 0.07, 0.66 | -0.023, 0.89 | 0.09, 0.57 |
| Positive P1 P3 G9 | 0.26, 0.10 | 0.27, 0.085 | 0.17, 0.28 |

**Supplementary Table 2. Correlations between clinical and demographic data and EEG data.** Table of Spearman correlations between delta ITC, theta ITC, and P3b peak amplitude and age, neuroleptics equivalent dose (mg/day), PANSS total positive, PANSS total negative, PANSS total general, PANSS total global, disorganized P2 N5 G10 G11, disorganized P2 P4 N5 N7, negative N1 G7 G13 G16, and positive P1 P3 G9.
